# Anti-inflammatory effects of shikonin in human periodontal ligament cells

**DOI:** 10.1080/13880209.2018.1506482

**Published:** 2018-11-04

**Authors:** Chen Fan, Xufang Zhang, Zee Upton

**Affiliations:** a Tissue Repair and Regeneration Program, Institute of Health and Biomedical Innovation, Queensland University of Technology, Brisbane, Australia;; b Institute of Medical Biology, Agency for Science, Technology and Research (A*STAR), Singapore;; c Skin Research Institute of Singapore, A*STAR, Singapore;; d Guangdong Province Key Laboratory of Stomatology, Department of Operative Dentistry and Endodontics, Guanghua School of Stomatology, Sun Yat-sen University, Guangzhou, People's Republic of China

**Keywords:** Inflammation, periodontitis, lipopolysaccharide, interleukin, tumor necrosis factor-α, matrix metalloproteinase-2, cyclooxigenase-2

## Abstract

**Context:** Shikonin (SHI), an active component extracted from *Radix Arnebiae*, has been reported to possess anti-inflammatory properties in various cells. However, its effect on lipopolysaccharide (LPS)-stimulated human periodontal ligament cells (hPDLCs) is unknown.

**Objective**: To investigate the effects of SHI on the expression of inflammatory related cytokines in LPS-stimulated hPDLCs.

**Materials and methods:** The effects of SHI (0.125, 0.25, 0.5, 1, and 2 μg/mL) on hPDLCs proliferation for 1, 3 and 7 days were measured using 3-(4,5-dimethylthiazol-2-yl)-2,5-diphenyltetrazolium bromide (MTT) assay. The expression of interleukin-1 (IL-1), IL-6, tumor necrosis factor-α (TNF-α), matrix metalloproteinase-2 (MMP-2), MMP-9 and cyclooxygenase-2 (COX-2) were detected in hPDLCs following SHI treatment (0.25 and 0.5 μg/mL) using Quantitative Reverse Transcriptase Polymerase Chain Reaction (qRT-PCR). The signaling pathways triggered by SHI in hPDLC were evaluated using western blotting.

**Results:** LD50 of SHI is 1.7 μg/mL (day 1) and 1.1 μg/mL (day 3 and 7) in hPDLCs. No morphological changes were observed when hPDLCs were treated with LPS only (1 μg/mL) or LPS with SHI (0.25 and 0.5 μg/mL). Data from qRT-PCR suggests that SHI attenuates LPS-induced increases of IL-1, IL-6, TNF-α, MMP-2, MMP-9 and COX-2 in hPDLCs. Down-regulation of phosphorylated extracellular signal-regulated kinase (ERK) and nuclear factor-κB (NF-κB), and up-regulation of I-κB, were observed in LPS-stimulated hPDLCs after exposed to SHI at 0.25 or 0.5 μg/mL.

**Discussion and conclusions:** SHI possesses anti-inflammatory effects in LPS-stimulated hPDLCs via phospho-ERK and NF-κB/I-κB signaling pathways; this suggests that SHI may hold potential as an anti-inflammatory agent against periodontitis.

## Introduction

Periodontitis is a chronic inflammatory disease caused by the accumulation of Gram-negative bacteria and results in periodontal tissue destruction and tooth loss (Andrukhov et al. 2013). LPS, a component existing in the cell walls of Gram-negative bacteria (Jang et al. 2011), has been demonstrated to induce the secretion of various inflammatory cytokines, such as IL-1 and IL-6 (Kato et al. 2014). hPDLCs have been found to play essential roles in periodontitis progression, as well as in the regeneration of periodontal tissues (Liu et al. 2017). In response to LPS, resident hPDLCs produce a range of cytokines, such as IL-1, TNF-α, MMP-2 and MMP-9, which are responsible for activation of circulating immune cells, destruction of collagen and resorption of alveolar bone (Shu et al. 2008). To date, conventional therapies for periodontitis, such as scaling and root planning, are widely used clinically (Qadri et al. 2015), however, these treatments are not effective in severe cases of periodontitis (Gonçalves et al. 2014). The investigation of novel anti-inflammatory agents is therefore essential to enable the development of improved and therapeutically effective treatments for periodontitis.

**Figure 1.**
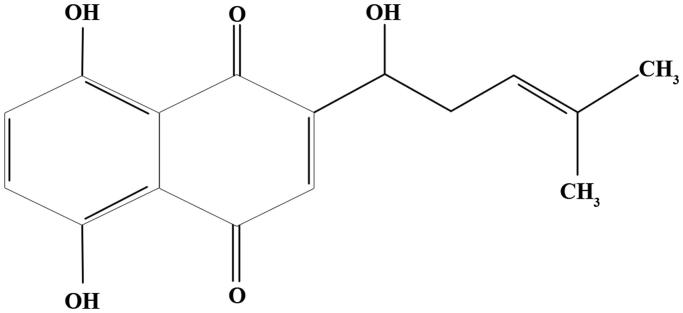
Chemical structure of shikonin (Zhao et al. 2017).

SHI (C_16_H_16_O_5_, Figure 1), an active component extracted from *Radix Arnebiae*, has been widely reported to possess anti-inflammatory properties (Fu et al. 2016). It has been reported that SHI prevents Ultraviolet B (UVB)-induced inflammation in keratinocytes by down-regulating IL-1, IL-6 and TNF-α expression (Ishida and Sakaguchi 2007). In addition, the expression of IL-4, IL-5 and TNF-α was attenuated in bone marrow-derived dendritic cells after SHI treatment (Lee et al. 2010). However, the mechanism of SHI anti-inflammatory effects on periodontitis-related cells is not known. Therefore, in the study reported herein, we investigated the mechanism of the anti-inflammatory effects of SHI in hPDLCs.

## Materials and methods

### Preparation of SHI treatment

SHI powder was produced by the National Institute for the Control of Pharmaceutical and Biological Products, China. SHI was dissolved in dimethyl sulfoxide (DMSO; 1.10 g/mL; Sigma-Aldrich, Sydney, Australia) as a stock solution and stored at −20 °C.

### Isolation and cultivation of hPDLCs

hPDLCs were obtained from consenting donors with ethics approval from the Queensland University of Technology (Human Ethics Committee 1300000144). The periodontal ligament was isolated from the middle third of the root surface following previously published methods (Qian et al. 2010). Cells were cultured in low glucose Dulbecco’s Modified Eagle Medium (DMEM; Life Technologies, Victoria, Australia) containing 10% (v/v) fetal bovine serum (FBS; In Vitro, Victoria, Australia) and 1% (v/v) penicillin/streptomycin (P/S; Life Technologies) at 37 °C in an incubator with 5% CO_2_.

### MTT assay

The effect of SHI on hPDLC proliferation was determined using the MTT assay. hPDLCs (3 × 10^3^ cells/well) were seeded into 96-well plates for 12 h. Cells were then treated with different concentrations of SHI (0.125, 0.25, 0.5, 1 and 2 μg/mL) for 1, 3 and 7 days, after which the cells were incubated with MTT solution (0.5 mg/mL, Sigma-Aldrich) at 37 °C for 4 h. Formazan crystals were then dissolved in DMSO (Sigma-Aldrich) and the absorbance was measured at 495 nm using a SpectraMax Microplate Reader (Molecular Devices, California, USA).

### FITC staining

Morphological changes in hPDLCs following SHI treatment were observed using FITC staining. hPDLCs were pretreated with SHI (0.25 and 0.5 µg/mL) for 1 h and then stimulated with LPS (1 µg/mL, dissolved in culture medium, Sigma-Aldrich) for 12 h. The hPDLCs were then fixed with 4% paraformaldehyde (PFA) for 30 min, prior to permeabilization with 0.2% Triton X-100/PBS for 5 min. The cells were then further incubated with 0.5% BSA/PBS containing 0.8 U/mL FITC (Sigma-Aldrich) and 5 µg/mL 4′,6-diamidino-2-phenylindole (DAPI; Sigma-Aldrich) for 1 h. Cell morphology was first recorded under light microscopy (Nikon Eclipse TS100, Nikon, Sydney, Australia) and then the fluorescence images were captured using a Nikon Eclipse TE2000-U microscope (Nikon).

### qRT-PCR

SHI-induced changes in hPDLC gene expression were determined using qRT-PCR. Primers for the target genes are listed in Table 1. The hPDLCs were pretreated with SHI (0.25 and 0.5 µg/mL) for 1 h and then stimulated with LPS (Sigma-Aldrich) for 1 and 3 days. Total RNA was then collected and extracted using TRIzol® Reagent (Life Technologies). First strand cDNA was synthesized using a DyNAmo™ cDNA Synthesis Kit (Life Technologies), according to the manufacturer’s protocol. qRT-PCR was performed using SYBR reagent in an ABI 7500 Thermal Cycler (Applied Biosystems, Victoria, Australia).

**Table 1.** Primers used in qRT-PCR.

Gene	Primers: Forward (F) and Reverse (R)	Annealing Temperature
IL-1	F: 5′-TTACAGTGGCAATGAGGATGAC-3′	60 °C
	R: 5′-TGCTGTAGTGGTGGTCGGAGA-3′	
IL-6	F: 5′-AGGAGACTTGCCTGGTGAAA-3′	58 °C
	R: 5′-CAGGGGTGGTTATTGCATCT-3′	
TNF-α	F: 5′-CCTGGTATGAGCCCATCTATC-3′	59 °C
	R: 5′-GGTTGGATGTTCGTCCTCCTC-3′	
MMP-2	F: 5′-CCGTCGCCCATCATCAA-3′	58 °C
	R: 5′-AGATATTGCACTGCCAACTCT-3′	
MMP-9	F: 5′-TCGTGGTTCCAACTCGGTTT-3′	61 °C
	R: 5′-GCGGCCCTCGAAGATGA-3′	
COX-2	F: 5′- AGGCTTCCATTGACCAGAGC-3′	60 °C
	R: 5′- TCCACAGCATCGATGTCACC-3′	
18s	F: 5′-TTCGGAACTGAGGCCATGAT-3′	60 °C
	R: 5′-CGAAC CTCCGACTTCGTTC-3′	

### Western blotting

SHI-induced protein changes in hPDLCs were detected using western blotting. hPDLCs were treated as described above and whole cell lysates were collected in a lysis buffer (Sigma-Aldrich) at 30 and 60 min after the treatment. The protein concentration was determined using the Bicinchoninic Acid Assay (BCA; Thermo Fisher Scientific, Victoria, Australia). Equal amounts of protein were prepared and separated using 10% sodium dodecyl sulfate polyacrylamide gel electrophoresis (SDS-PAGE) and were then transferred onto nitrocellulose membranes (Pall Corporation, New York, USA). The membranes were incubated with primary antibodies overnight at 4 °C in Odyssey blocking buffer (LI-COR^®^ Biosciences, Northeastern, USA). Primary antibodies included: ERK1/2, p-ERK1/2, c-Jun N-terminal kinase (JNK)1/2, p-JNK1/2, NF-κB and I-κB from Genesearch, Queensland, Australia; mitogen-activated protein kinase 14 (p38α/β) and p-p38α/β from Santa Cruz Biotechnology, Texas, USA; and GAPDH from Sigma-Aldrich. The membranes were then incubated with corresponding fluorescent secondary antibodies (Genesearch). The images were captured and analyzed using the Odyssey Imaging system and software (LI-COR^®^ Biosciences).

### Statistical analysis

Samples were collected from three different patients. The treatment and all assays were performed in triplicate. All data were converted to the percentage of the control (no SHI treatment) and One-way ANOVA and Tukey’s *post hoc* test was used to analyze the statistical difference. *p* < 0.05 was regarded as statistically significant.

## Results

### Effects of SHI on hPDLCs proliferation

**Figure 2.**
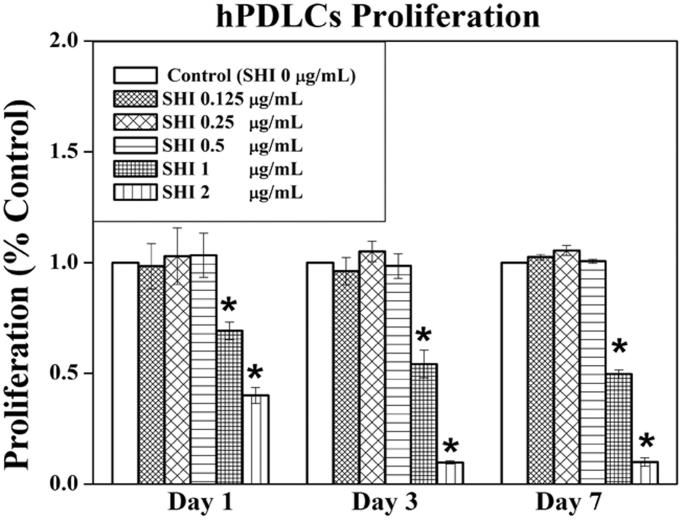
Effect of SHI on hPDLC proliferation. hPDLCs were exposed to SHI (0.125, 0.25, 0.5, 1, and 2 μg/mL) for 1, 3 and 7 days. Cell proliferation was measured using the MTT assay. The data are expressed as the percentage of the control (containing medium only). Error bars indicate mean ± SEM (*n* = 3). *p < 0.05 versus the control. Statistical analysis was performed using One-way ANOVA and Tukey’s *post hoc* test.

To evaluate the effects of SHI on cell proliferation, hPDLCs were treated with different concentrations of SHI (0.125, 0.25, 0.5, 1, and 2 μg/mL) for 1, 3 and 7 days (Figure 2). As shown in Figure 2, no differences in the effects of SHI at 0.125, 0.25 and 0.5 μg/mL on hPDLC proliferation were observed compared to the control (*p* > 0.05). However, SHI at 1 and 2 μg/mL significantly inhibited hPDLC proliferation at day 1, 3 and 7 compared to the control (*p* < 0.05). Taken together, these data suggest that SHI inhibits hPDLC proliferation in a dose-dependent manner, notwithstanding that SHI at 0.125, 0.25 and 0.5 μg/mL has no observable effect on hPDLC proliferation.

### Morphology of hPDLCs following LPS and SHI treatment

**Figure 3.**
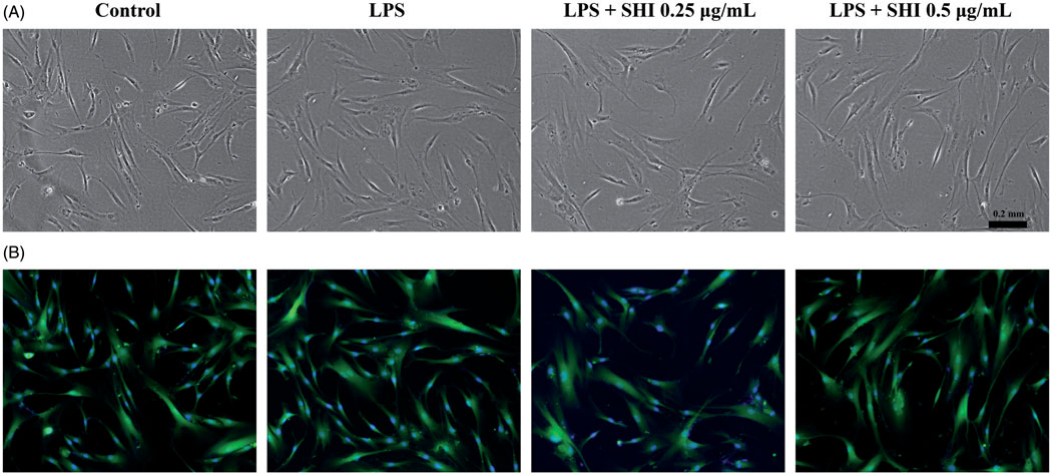
Effect of SHI on hPDLC morphology. hPDLCs were pretreated with SHI (0.25 and 0.5 µg/mL) for 1 h and then stimulated with LPS for 12 h. Images were then captured using a Nikon Eclipse TS100 light microscope (A) and a Nikon Eclipse TE2000-U fluorescence microscope (B).

Cell morphology after treatment with SHI and LPS was first recorded using light microscopy (Figure 3(A)) and then further images were captured using fluorescence microscopy (Figure 3(B)) after probing with FITC and DAPI. As shown in Figure 3, no effects of LPS or LPS with SHI (0.25 and 0.5 μg/mL) on hPDLC morphology were observed.

### Anti-inflammatory effect of SHI on hPDLCs

**Figure 4.**
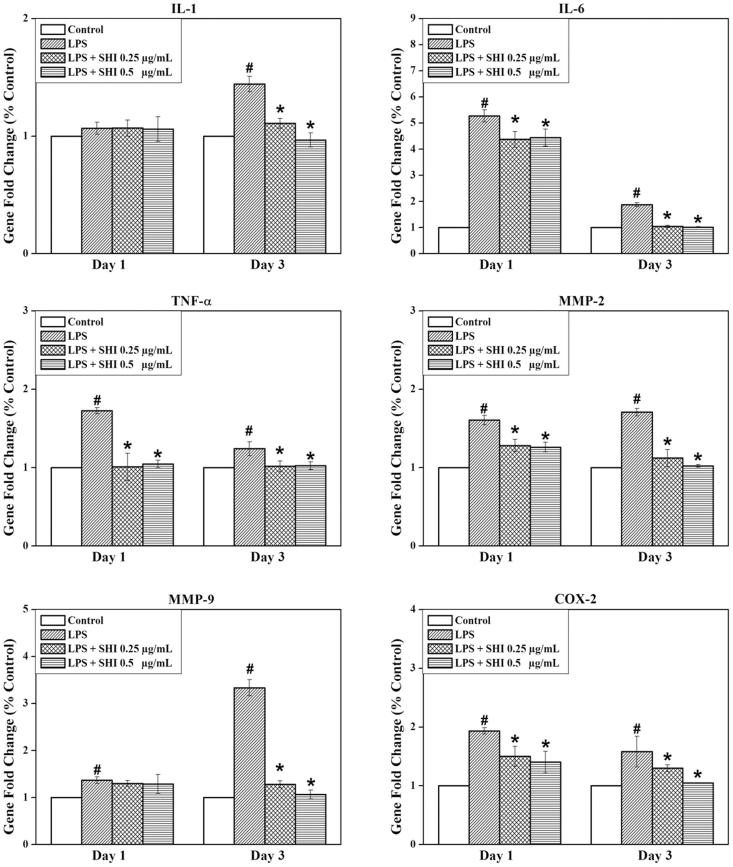
Effect of SHI on hPDLC gene expression. hPDLCs were pretreated with SHI (0.25 and 0.5 µg/mL) for 1 h and then stimulated with LPS for 1 and 3 days, after which total RNA was collected. After RNA extraction, first strand cDNA was synthesized. The cDNA sample was then amplified using qRT-PCR. The expression of the target gene was first normalized to 18s and then further converted to the percentage of the control. Error bars indicate mean ± SEM (*n* = 3). #*p* < 0.05 versus the control while *p < 0.05 versus the LPS group. Statistical analysis was performed using One-way ANOVA and Tukey’s *post hoc* test.

To identify the anti-inflammatory effect of SHI on hPDLCs, expression of genes for IL-1, IL-6, TNF-α, MMP-2, MMP-9 and COX-2 were investigated using RT-PCR (Figure 4). The expression of all of these genes was found to be greater than the control when the cells were exposed to LPS for either 1 or 3 days (*p* < 0.05), except IL-1 showed non-significant increase after 1 day. However, pretreatment with SHI at 0.25 and 0.5 μg/mL significantly attenuated the expression of all these genes at day 3 compared to the control and LPS-simulated cells (*p* < 0.05). In addition, IL-6, TNF-α, MMP-2 and COX-2 were found to be down-regulated in hPDLC cells pretreated with SHI at 0.25 and 0.5 μg/mL prior to 1 day of LPS stimulation compared to the control and LPS-simulated cells (*p* < 0.05). In summary, these results indicate that pretreatment with SHI inhibits inflammation-related gene expression in hPDLCs following stimulation with LPS.

### SHI-induced signaling pathways in hPDLCs

**Figure 5.**
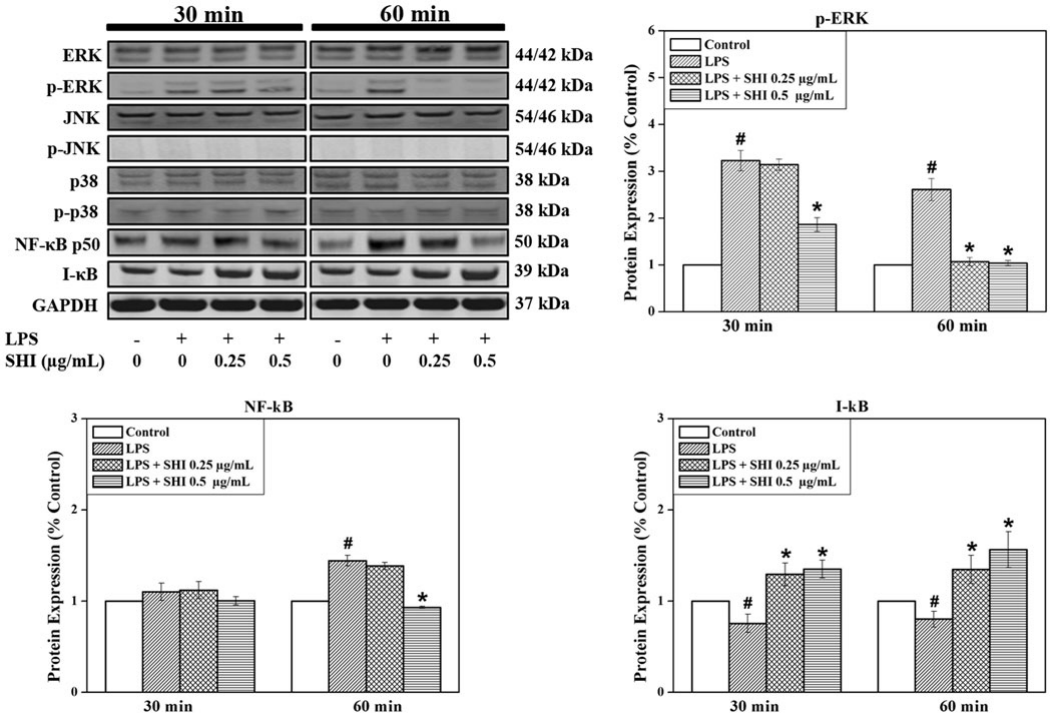
SHI-induced signaling pathways in hPDLCs. hPDLCs were pretreated with SHI (0.25 and 0.5 µg/mL) for 1 h and then stimulated with LPS for 30 and 60 min, after which, total protein was collected. The levels of protein expression were determined by western blotting. The expression of p-ERK was converted to the percentage of total ERK; expression of NF-κB and I-κB were converted to GAPDH. Then the data was further converted into the percentage of the control group. Error bars indicate mean ± SEM (*n* = 3). #*p* < 0.05 versus the control while *p < 0.05 versus the LPS group. Statistical analysis was performed using One-way ANOVA and Tukey’s *post hoc* test.

The effects of pretreatment with SHI on the expression of ERK, JNK, p-38, NF-κB and I-κB in hPDLCs were also evaluated since these signaling molecules play vital roles in mediating the inflammation process (Chen et al. 2013; Guo et al. 2013). As demonstrated in Figure 5, no effects of SHI on ERK, JNK, p-JNK, p-38 and p-p38 were observed in cells exposed to SHI prior to stimulation with LPS. However, the expression of p-ERK was observed to be significantly down-regulated when the cells were exposed to SHI at 0.25 and 0.5 μg/mL prior to stimulation with LPS for 60 min, compared to LPS-stimulated cells (*p* < 0.05). Reduced p-ERK expression was also observed when the cells were pretreated with SHI with 0.5 μg/mL prior to LPS stimulation for 30 min. In addition, SHI at 0.5 μg/mL attenuated NF-κB expression at 60 min compared to LPS-stimulated cells (*p* < 0.05). Furthermore, SHI at 0.25 and 0.5 μg/mL significantly up-regulated I-κB expression at 30 and 60 min compared to LPS-stimulated cells (*p* < 0.05). In summary, these data illustrate that SHI may inhibit the inflammatory responses in hPDLCs following exposure to LPS by reducing p-ERK and NF-κB and increasing I-κB expression.

## Discussion

Bacteria-induced inflammation is an important pathological condition in periodontitis (Darveau 2010). Numerous studies have focused on the role of hPDLCs in periodontitis as they secret inflammatory mediators in response to bacterial pathogens (Wada et al. 2004). SHI is an active component extracted from *Radix Arnebiae* and possesses anti-inflammatory properties (Fu et al. 2016). Previous study has shown that SHI inhibits the inflammatory responses in IL-1β- and TNF-α-stimulated hPDLCs (Shindo et al. 2016), suggesting the potential use of SHI as an anti-inflammatory agent against periodontitis. In the current study, we further evaluated the potential medical value of SHI using LPS-stimulated hPDLCs.

The importance of inflammatory cytokines in periodontitis has been widely documented. During the development of periodontitis, over-expression of IL-1, IL-6 and TNF-α leads to the loss of periodontal attachment (Gemmell et al. 1997). Elevated levels of IL-1, IL-6 and TNF-α have been found in the gingival crevicular fluid from patients with periodontitis (Rossomando et al. 1990; Geivelis et al. 1993; Ishihara et al. 1997). IL-1 and TNF-α antagonists have been shown to inhibit the inflammatory response and bone destruction in experimental periodontitis (Assuma et al. 1998; Delima et al. 2001). MMP-2 and MMP-9 are known to be involved in digestion of bone matrix (Makela et al. 1994; Sorsa et al. 2006). Indeed, the levels of MMP-9 in gingival crevicular fluid have been used to diagnose the progression of periodontitis (Sorsa et al. 2006). COX-2 is also known to contribute to the development of periodontitis as it is a key mediator maintaining vascular homeostasis (Mendes et al. 2014). SHI has been previously reported to down-regulate IL-1 and TNF-α expression in the joints of arthritic mice (Kim et al. 2010). It is also reported that SHI inhibits tumor invasion by reducing MMP-9 expression in human adenoid cystic carcinoma cells (Min et al. 2011). Moreover, expression of COX-2 in experimental colitis has been shown to be attenuated following SHI treatment (Andújar et al. 2012). Consistent with previous studies, our results indicate that SHI down-regulates IL-1, IL-6, TNF-α, MMP-2, MMP-9 and COX-2 expression in LPS-stimulated hPDLCs, thus indicating the anti-inflammatory potential of SHI in the treatment of periodontitis.

NF-κB is regarded as the master regulator of inflammation and host immune responses. It is known that NF-κB pathways are essential for LPS-induced production of inflammatory cytokines and MMPs, including IL-1, IL-6, TNF-α, MMP-2 and MMP-9 (Yip et al. 2004; Yan and Boyd 2007). For example, the expression of IL-1, IL-6 and TNF-α have been demonstrated to be mediated via the activation of the NF-κB signaling pathway in H9c2 cardiac cells (Guo et al. 2013). Under normal conditions, NF-κB, a heterodimer composed of two subunits (p50 and p65), is bound to its inhibitor, I-κB, and resides in the cytoplasm as an inactive NF-κB/I-κB complex (Napetschnig and Wu 2013). After I-κB is phosphorylated, it is degraded by ubiquitination, allowing NF-κB to translocate to the nucleus (Hosokawa et al. 2013). Our results demonstrated that SHI inhibits the NF-κB signaling pathway in LPS-stimulated hPDLCs via decreasing NF-κB p50 activation and I-κB degradation. Thus, it is plausible that the down-regulation of inflammatory cytokines and MMPs that we observed in hPDLCs following treatment with SHI may be mediated by the inhibitory effect of SHI on NF-κB activation. In our previous publication, SHI was found to increase the phosphorylation of NF-κB and reduce the phosphorylation of I-κB in human dermal fibroblasts (Fan et al. 2015). Interestingly, we did not observe the equivalent response to SHI on the phosphorylation of I-κB in hPDLCs in this study. This suggests that SHI may regulate the NF-κB/I-κB signaling pathway via different ways in different cell types. Therefore, effects of SHI on the phosphorylation of I-κB, as well as the translocation of NF-κB, need to be further investigated.

In addition to responding to extracellular stimulators, the activation of NF-κB can also be initiated by intra-cellular kinases, such as mitogen-activated protein kinases (MAPKs). MAPKs, including ERK, JNK and p-38, are broadly reported to participate in the regulation of inflammatory responses. It has been demonstrated that phosphorylation of ERK is an upstream signal, necessary for the activation of the NF-κB signaling pathway (Filip et al. 2013). ERK inhibitors, but not JNK or p38 MAPK inhibitors, have been shown to attenuate force-mediated stimulation of NF-κB-DNA binding in hPDLCs (Kook et al. 2011), indicating the importance of ERK signaling in periodontitis and its correlation with NF-κB. Further, other reports indicate that IL-1 and IL-6 are regulated by the activation of both ERK and NF-κB signaling pathways in macrophages (Choi et al. 2013). It has been reported that LPS activates the phosphorylation of MAPK family proteins, including ERK, JNK and p-38. It has also been shown that LPS significantly activates the phosphorylation of ERK, JNK and p38 in murine bone marrow macrophages (Hashimoto et al. 2017). However, the phosphorylation of MAPK proteins varies in different types of cells or tissues. For example, Zhang et al. (2017) reported that LPS significantly activates the phosphorylation of ERK and p-38 in mice, however, no phosphorylation of JNK was observed. LPS has been found to significantly enhance the phosphorylation of ERK and JNK in RAW264.7 cells, but only a slight effect on phosphorylation of p-38 was seen (Jang et al. 2011). Further, when LPS is mixed with Nicotine, a significant increase in the phosphorylation of JNK and p-38 in hPDLCs is observed, however, no effect on the expression of phosphorylated ERK was detected (Jeong et al. 2009). Therefore, the LPS-triggered signaling pathways in hPDLCs need to be further evaluated. In our study, no effects of SHI on the expression of JNK and p-38 were observed in hPDLCs. However, attenuation of p-ERK and NF-κB p50 and increases in I-κB (inhibitor of NF-κB) were observed in hPDLCs following SHI treatment. These data indicate that the SHI-induced anti-inflammatory effects in hPDLCs are related to the inhibition of the ERK and NF-κB signaling pathways.

In conclusion, SHI exhibits anti-inflammatory effects in LPS-stimulated hPDLCs *in vitro*. The mechanisms for the inhibitory effects of SHI on inflammatory processes in hPDLCs may be related to the suppression of NF-κB/I-κB and ERK signaling pathways. We therefore propose that SHI has the potential to be utilized as a supplement to current therapeutic regimens addressing periodontitis.
